# Longitudinal analysis shows possible distinct patterns of associations between conspiracy beliefs and either institutional distrust or the sense of precarity

**DOI:** 10.1111/bjso.70036

**Published:** 2025-12-15

**Authors:** Magdalena Adamus, Jakub Šrol, Eva Ballová Mikušková, Jais Adam‐Troian, Maria Chayinska

**Affiliations:** ^1^ Faculty of Economics and Administration Masaryk University in Brno Brno Czechia; ^2^ Centre of Social and Psychological Sciences Slovak Academy of Sciences Bratislava Slovakia; ^3^ School of Psychology Heriot‐Watt University Dubai UAE; ^4^ Department of Cognitive, Psychological and Pedagogical Sciences, and Cultural Studies University of Messina Messina Italy

**Keywords:** AR‐CLPM, conspiracy beliefs, institutional trust, longitudinal analysis, precarity, RI‐CLPM

## Abstract

The paper reports longitudinal analyses examining the extent to which institutional trust mediates the relationship between individuals' sense of precarity and their adherence to conspiracy beliefs. Across three waves, 925 participants (50.2% female) between the ages of 18 and 85 (M = 49.53; SD = 15.81) reported subjective appraisals of their financial situation (precarity), trust in institutions and adherence to conspiracy beliefs. The current study extends the previous analyses by including three‐wave longitudinal data. The preregistered autoregressive cross‐lagged panel model supports the notion that a sense of precarity follows adherence to conspiracy beliefs rather than preceding them, while institutional (dis)trust and conspiracy beliefs show a bidirectional pattern. However, the random‐intercept cross‐lagged panel model does not corroborate this, suggesting that the effects may be driven by stable between‐person differences rather than actual within‐person changes. Additionally, the latter model reveals two separate temporal patterns linking conspiracy beliefs with either the sense of precarity or institutional trust, opening the possibility that our results were driven by two distinct underlying mechanisms. The paper discusses the importance of longitudinal studies for a more accurate understanding of social‐psychological realities in which conspiracy beliefs and suspicions of institutions may flourish.

## INTRODUCTION

Previous research has suggested that individuals from lower social classes are more susceptible to endorsing *conspiracy theories* that are understood to suggest straightforward explanations for distressing and complex phenomena as concocted by powerful and malicious groups (Mao et al., 2020; Uscinski & Parent, 2014). Recently, however, it has been proposed to disentangle and quantify the respective impacts of *poverty* (i.e., the state of being inferior in terms of a usual or socially acceptable amount of money) and *precarity* (i.e., subjective appraisal of one's living circumstances as insufficient and threatening), as these two constructs might have different effects on adherence to conspiracy beliefs (CBs) in the general population (Standing, 2011). Indeed, the research we attempt to extend in the current study has documented that subjective appraisals of one's own financial situation as precarious were likely to be even more important a factor – compared to objective income or traditional class considerations – in enhancing an individual's adherence to CBs (Adam‐Troian et al., 2023; Adamus et al., 2024).

Offering some preliminary evidence for this notion, the two studies explained that experiences of precarity are likely to evoke doubts, suspicion and distrust, all of which have been previously identified as precursors of CBs (Meuer & Imhoff, 2021; Pummerer, 2022; Pummerer et al., 2022). Relatedly, the experience of precarity was shown to have numerous adverse impacts on one's quality of life (e.g., mental health, sleep deprivation, chronic pain or malnutrition; Bjorklund, 2023) and cognitive functions (e.g., memory, executive control and analytical thinking), thus making individuals more prone to adhere to CBs (de Bruijn & Antonides, 2020; Fiksenbaum et al., 2017; Haushofer & Fehr, 2014; Mani et al., 2013; Mullainathan & Shafir, 2013). It was further shown that people's susceptibility to various epistemically suspect beliefs (including CBs) that offer seemingly logical explanations for complex events serves them as a defensive and coping strategy in alleviating cognitive dissonance and reducing feelings of discomfort, threat and fear (Bukowski et al., 2017; Johnson‐Schlee, 2019; Jolley & Paterson, 2020; Kraus et al., 2012; Marchlewska et al., 2017). Importantly, apart from any debilitating effects precarity may have on cognition, people who live in precarious conditions may have deeply objectionable experiences with institutions and their representatives and thus have real and rational reasons to become suspicious of political elites and institutions, whom they hold accountable for the hardship they experience (Cottrell & Neuberg, 2005; Jovančević & Milićević, 2020; Kraus et al., 2012; Wagner‐Egger et al., 2022). Therefore, it does not come as a surprise that not only is institutional trust (or lack thereof) consistently associated with adherence to CBs (Meuer & Imhoff, 2021; Pummerer, 2022; Pummerer et al., 2022), but it mediates the relationship between precarity and adherence to CBs as well (Adam‐Troian et al., 2023; Adamus et al., 2024).

While both the original study (Adam‐Troian et al., 2023) and its conceptual replication (Adamus et al., 2024) used large international samples and delivered persuasive findings that contributed to our knowledge of how institutional trust and the sense of precarity are associated with adherence to CBs, the data they both analysed were cross‐sectional. As a consequence, our knowledge about those patterns of relationships remains to a large extent correlational. Generally, there is still relatively little research indicative of causal and temporal patterns of relationships between CBs and their potential social‐psychological causes and consequences. Therefore, unsurprisingly, the two original studies straightforwardly posited that the sense of precarity is an antecedent of an enhanced adherence to CBs, while institutional trust serves as a mediator. To disentangle the relationships and delve deeper into the temporal sequences, we would need experimental and longitudinal research on the phenomena in question. Among the few available studies that have attempted a longitudinal investigation, it is worth mentioning recent literature arguing that the relationship between people's distrust in political institutions and adherence to CBs is, in all likelihood, bidirectional (van Prooijen et al., 2022) – a pattern that can hardly be observed cross‐sectionally. This suggests that low institutional trust and CBs may be closely associated in a vicious circle in which the negative effects of the two phenomena reinforce one another. Moreover, social‐psychological research shows that the more people believe they are targets of injustice or nefarious plots, the more likely they are to experience anxiety, delusions and depression (Ascone et al., 2017; Chayinska & Minescu, 2018; Grupe & Nitschke, 2013; Mathews & MacLeod, 1985). From this perspective, the sense of victimization, the interpretation of events as malicious schemes and the associated persecutory delusions may lead to elevated levels of anxiety, including the sense of economic anxiety (Adamus et al., 2025; Clarke et al., 2018; Eysenck et al., 1991; Gagliardi, 2023; Lam et al., 2021; Liekefett et al., 2023; Mathews & MacLeod, 1985; Salemink et al., 2009; Trotta et al., 2021).

The above findings raised our first doubts about whether cross‐sectional results can provide a comprehensive picture of the social‐psychological reality of CBs. Specifically, the current longitudinal reanalysis was directly inspired by recent findings about a phenomenon closely related to precarity – economic anxiety – persuasively claiming that instead of being an *antecedent*, economic anxiety is an *outcome* of adherence to CBs (Adamus et al., 2025). Rather than the other way around, adherence to CBs consistently led to more pessimistic appraisals of the country‐level and individual economic situations. It thus seems conceivable that the gloomy appraisal of the economic and institutional environment that follows adherence to CBs may also include conspiracy theorists' assessment of their own economic situation and an aggravated sense of living in financially precarious circumstances. The results inspired us to reconsider the previously established mediation model in an attempt to disentangle the intrinsic temporal sequences of the phenomena – institutional trust, precarity and CBs – instead of capturing momentary snapshots of the cross‐sectional status quo. The present study thus responds directly to the call for longitudinal studies to establish whether a change in one's precarious living circumstances or perceived trustworthiness of institutions would be followed by changes in adherence to CBs.

## METHODS

### Participants

Data were collected through an online survey hosted in Qualtrics. The survey was administered as part of a larger three‐wave longitudinal study mapping the social‐psychological consequences of the COVID‐19 pandemic. A total of 925 adults (50.2% female) between the ages of 18 and 85 (M = 49.53; SD = 15.81) took part in all three data collection waves. In terms of education in the final sample, 26.4% of participants had attained an elementary or incomplete grammar school education; 48.5% had completed grammar school, and 25.1% had either higher education or completed college. The data were collected in October 2021 (Wave 1), July–August 2022 (Wave 2) and April–May 2023 (Wave 3). Regarding attrition, 1838 participants took part in the first wave of the data collection, and 1426 took part in the second wave of the data collection, representing 22.4% attrition between the first two waves and 35.1% attrition between the second and the third wave of the data collection. With regard to the basic demographic variables, the original sample in the first wave was slightly younger (M = 45.46; SD = 16.12), had a higher representation of women (52.7%) and included participants with lower levels of education (35.0% with elementary or incomplete grammar school, 43.4% with completed grammar school and 21.7% with either higher education or completed college) than the final sample analysed in the present study.

We have also calculated an attrition check to see whether participants with certain characteristics (institutional trust, precarity and conspiracy beliefs, as well as key demographic variables) were more likely to continue participation across waves. These results are presented in Section A of the Data S1. As can be seen there, both higher age and education were positively associated with the number of waves completed, suggesting our final sample underrepresents younger and less educated participants. However, and crucially, neither of the three key variables tested in our main models was significantly associated with continued participation across waves. Therefore, the main conclusions of the mediation models presented below are not driven by participant attrition. Participants were recruited from an online panel by a research agency in accordance with ESOMAR standards. Compensation for participation was determined by the agency's internal scoring system. The sample was balanced in terms of age, gender, region and education.

### Transparency and openness

The study reports a secondary analysis of longitudinal data collected during a three‐wave mini‐longitudinal project in Slovakia between October 2021 and May 2023 (see https://osf.io/28vs4/overview and https://osf.io/pqk9z/overview for details). The data used in this study are part of a larger project and have already been analysed for other research purposes. Specifically, the data about the sense of financial threat, institutional trust and CBs were analysed separately for the first and second waves in Adamus et al. (2024) and Merva et al. (2025). The financial threat scale was also used as a component of an economic anxiety index for an analysis in Adamus et al. (2025). The current study was registered as an exploratory analysis of a longitudinal mediation model that attempts to track and establish the directionality of relationships between the three variables in question (see OSF for the registration details: https://osf.io/kgc4f/overview). All materials, raw data and analysis scripts used in this study are available at: https://osf.io/pqk9z/overview.

### Measures

#### Precarity

Precarity was measured using six items from the modified version of the Financial Threat Scale (FTS) asking participants to indicate how they feel about their current financial situation (e.g., ‘How uncertain do you feel?’) (Marjanovic et al., 2015). Participants were required to indicate how they rated the stability and security of their personal finances during the pandemic on a 5‐point scale (from 1 = not at all to 5 = very much). We used an average rating on the six items measured at every respective wave of the study as an indicator of precarity. The measure showed very high reliability at all three waves of data collection (T1: *α* = .91, T2: *α* = .94, T3: *α* = .93).

#### Conspiracy beliefs

Conspiracy beliefs were measured using 18 items from the COVID‐19 Unfounded Beliefs Scale (C19‐UB) (Teličák & Halama, 2022). Participants were asked to indicate their agreement with conspiracy beliefs related to COVID‐19 (5 items, for example, ‘COVID‐19 was planned long ago to weaken the economy and cause unemployment’), measures taken (4 items, for example, ‘Wearing protective masks is dangerous for schoolchildren and the elderly’), treatment (5 items, for example, ‘Intravenous (injectable) use of sodium chlorite has proven to be effective against COVID‐19’) and vaccinations (3 items, for example, ‘Vaccines against COVID‐19 contain substances that cause infertility or abortion’). Higher mean scores on the 18 items indicate higher endorsement of conspiracy theories (5‐point scale: from 1 = totally disagree to 5 = totally agree). The measure showed excellent reliability at all three waves of data collection (T1: *α* = .96, T2: *α* = .96, T3: *α* = .96).

#### Institutional trust

To capture institutional trust, we asked participants to what extent they personally trusted each of eight institutions or ten institutions (for example, the Ministry of Health of the Slovak Republic, the European Medicines Agency, the Slovak Academy of Sciences, doctors and health professionals) in the second and third waves (10‐point scale, from 1 = absolutely do not trust to 10 = absolutely trust). An average rating for the eight (first wave) or ten (second and third wave) institutions was used as an indicator of institutional trust. The measure showed excellent reliability at all three waves of data collection (T1: *α* = .94, T2: *α* = .96, T3: *α* = .95). Starting from the second wave, two additional items were added as relevant at that time. First, because of the conspiracy beliefs about COVID‐19 vaccines and treatments, we added an item about trust in scientists. Second, because the second wave was conducted after the Russian invasion of Ukraine, we also added an item about trust in the army. To ensure that our findings are not driven by differences in the measurement of institutional trust across the waves, we have also recalculated the main analyses below using just the subset of eight items consistent across all three waves. These results are available in Section B of the Data S1 and are completely consistent with the analyses presented in the main manuscript, therefore providing further evidence about the robustness of our conclusions.

## RESULTS

In accordance with the preregistration, we tested a three‐wave autoregressive cross‐lagged panel model (AR‐CLPM) with precarity, institutional trust and conspiracy beliefs. Based on previous results (Adam‐Troian et al., 2023; Adamus et al., 2024), our presumed mediation model included precarity as a predictor (X), institutional trust as a mediator (M) and conspiracy beliefs as the outcome (Y). However, we also tested alternative models to be able to identify the one with the best fit to our data. First, for comparison purposes, we estimated an autoregressive model with all paths between our three variables. Then, we tested our presumed mediation model, as well as a model with opposite mediation paths (X: conspiracy beliefs, M: institutional trust, Y: precarity) and a third alternative model (X: institutional trust, M: conspiracy beliefs, Y: precarity). We analysed the model fit through multiple indicators: the comparative fit index (CFI), the Tucker‐Lewis index (TLI) and the root mean square error of approximation (RMSEA). For these indices, values of CFI > 0.95, TLI > 0.95 and RMSEA < 0.06 were considered to indicate good model fit, and values of CFI > 0.90, TLI > 0.90 and RMSEA < 0.08 were considered to indicate acceptable model fit. Finally, we tested for differences in the model fit between the model with all estimated paths and the three constrained mediation models via a χ^2^ difference test. All models were estimated using the *lavaan* package (Rosseel, 2012) in R software. All variables included in the models were entered as observed variables, rather than latent constructs. For a more precise specification of our models, please see the attached analysis script available at: https://osf.io/pqk9z/overview.

**FIGURE 1:**
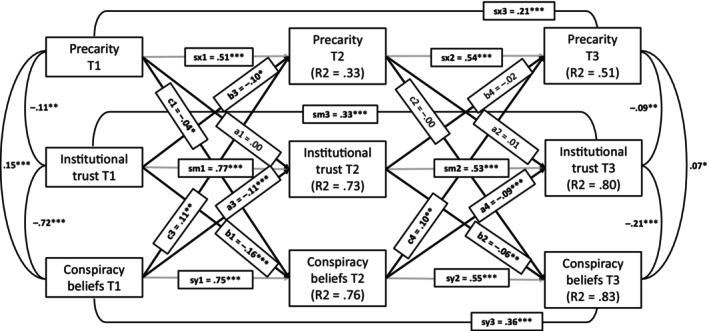
Autoregressive model with all paths between precarity, institutional trust and conspiracy beliefs estimated. The figure presents standardized estimates and their statistical significance. *p < .05, **p < .01, ***p < .001.

First, we estimated an autoregressive model with all paths between our three variables of interest (Figure 1). We selected the AR‐CLPM to control for the temporal stability of the variables included in our models that could otherwise lead to inflated coefficients for the associations between them. All indicators suggested that this model had a very good fit to the data, χ^2^(7) = 8.47, *p* = .29, CFI = 1.00, TLI = 0.99, RMSEA = 0.015, SRMR = 0.010. Importantly, as can be seen from the autoregressive paths in this model, while institutional trust and conspiracy beliefs are relatively stable over time, precarity is much more volatile between the waves.

**FIGURE 2:**
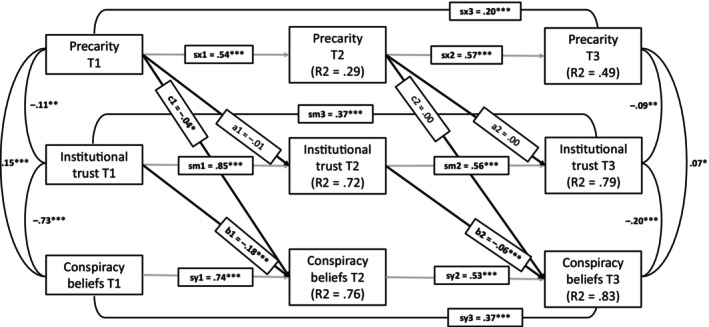
Autoregressive mediation model with only presumed paths between precarity, institutional trust and conspiracy beliefs estimated. The figure presents standardized estimates and their statistical significance. *p < .05, **p < .01, ***p < .001.

Next, we estimated the model with our presumed mediation paths (Figure 2). While CFI and TLI suggested a good fit for this model, the RMSEA value was higher than the threshold for an acceptable model fit, χ^2^(13) = 111.96, *p* < .001, CFI = 0.99, TLI = 0.96, RMSEA = 0.091, SRMR = 0.093. The model fit was also much worse in comparison with the model with all paths estimated, χ^2^(6) = 103.5, *p* < .001. Crucially, our presumed indirect longitudinal path (a1*b2) was not significant, estimate = 0.000, 95% CI [−0.002, 0.003], standardized = 0.000, *p* = .77. This was obviously because precarity did not significantly predict institutional trust longitudinally, even though institutional trust did predict adherence to conspiracy beliefs.

**FIGURE 3:**
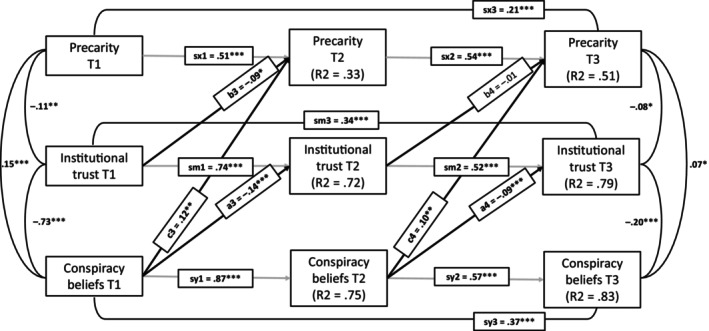
Autoregressive mediation model with only opposite to presumed paths between precarity, institutional trust and conspiracy beliefs estimated. The figure presents standardized estimates and their statistical significance. *p < .05, **p < .01, ***p < .001.

Therefore, we estimated an opposite model (X: conspiracy beliefs, M: institutional trust, Y: precarity, see Figure 3) to see whether it would have a better fit to our data. Indeed, while CFI and TLI again indicated a good fit of the model, RMSEA suggested at least an acceptable, but not good, model fit, χ^2^(13) = 71.15, *p* < .001, CFI = 0.99, TLI = 0.98, RMSEA = 0.070, SRMR = 0.028. However, the opposite model had a significantly worse fit in comparison with the model with all paths estimated, χ^2^(6) = 62.7, *p* < .001. Also, the opposite indirect longitudinal path (a3*b4) was, again, not statistically significant, estimate = 0.002, 95% CI [−0.007, 0.0100], standardized = 0.002, *p* = .68, because while conspiracy beliefs predicted institutional trust, trust did not predict increased precarity over time.

**FIGURE 4:**
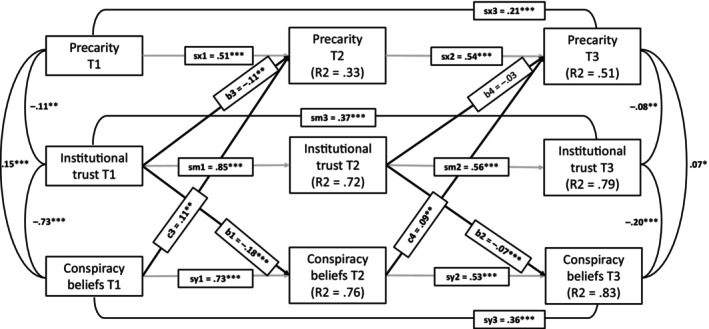
Autoregressive mediation model with only alternative paths between precarity, institutional trust and conspiracy beliefs estimated. The figure presents standardized estimates and their statistical significance. *p < .05, **p < .01, ***p < .001.

Finally, we tested a third alternative model (X: institutional trust, M: conspiracy beliefs, Y: precarity, see Figure 4). Surprisingly, according to all considered indicators, this model showed the best fit to the data among the examined models: χ^2^(13) = 48.30, *p* < .001, CFI = 0.99, TLI = 0.99, RMSEA = 0.054, SRMR = 0.023. While it still performed worse in comparison with the model presented in Figure 1, the difference in model fit was smaller than the one for the other two tested mediation models, χ^2^(6) = 39.8, *p* < .001. Also, crucially, the alternative indirect longitudinal path estimated in this model (b1*c4) was significant, estimate = −0.007, 95% CI [−0.012, −0.002], standardized = −0.015, *p* = .010. As can be seen from the figure, institutional trust negatively predicted adherence to conspiracy beliefs, and these, in turn, positively predicted precarity across all waves of our longitudinal data collection. However, the direct path from institutional trust to precarity was significant only between the first two waves. Using the benchmarks for effect sizes in autoregressive models suggested by Orth et al. (2024), the longitudinal associations are all of medium or large effect.

### Random‐intercept cross‐lagged panel model

Although not preregistered, we also tested a random‐intercept cross‐lagged panel model (RI‐CLPM) to separate the stable between‐person associations between precarity, trust and conspiracy beliefs from within‐person changes in these variables over time. The model, estimated using the R package *lavaan* (Rosseel, 2012) with standard errors based on 2000 bootstrap samples, showed a good fit to the data according to most of the indicators, χ^2^(3) = 30.2, *p* < .001, CFI = .99, TLI = .96, RMSEA = .099, SRMR = .027. While the value for RMSEA indicated a poor fit of our RI‐CLPM model, RMSEA has been shown to falsely indicate poor fit in models with low degrees of freedom (Kenny et al., 2015), such as ours. Therefore, the model was retained.

**FIGURE 5:**
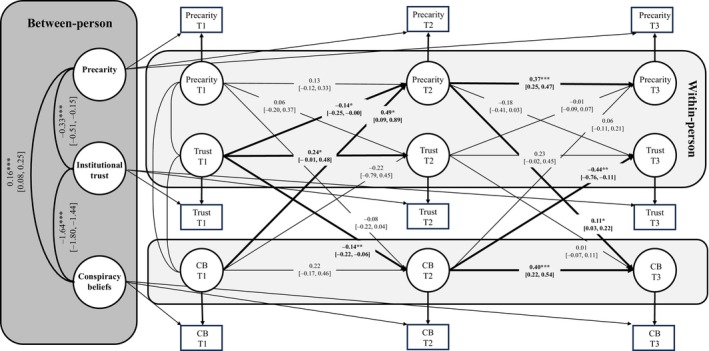
Random‐intercept cross‐lagged panel model of the relationships between precarity, institutional trust and conspiracy beliefs. Values for paths represent unstandardized coefficients along with their 95% confidence intervals. CB, conspiracy beliefs. Significant paths (*p* < .05) are presented in bold. *p < .05, **p < .01, ***p < .001.

As can be seen from Figure 5, at the between‐person level, the random intercepts for our three variables were significantly correlated. Specifically, completely standardized coefficients for the covariances showed that precarity was associated with lower trust (*r* = −.22) and higher belief in conspiracy theories (*r* = .24), and that the latter two variables were very strongly negatively correlated (*r* = −.79). On the level of within‐person associations, however, we mostly see some significant autoregressive associations showing that within‐person deviations from the individual's trait‐level mean at one wave persisted at the subsequent wave of the data collection. While between the first two waves of the data collection, we found a significant within‐person autoregressive effect only for institutional trust, precarity and conspiracy beliefs showed significant autoregressive effects between the second and the third wave of the data collection.

More importantly, we found that lower levels of institutional trust at the first wave predicted within‐person increases in both the sense of precarity and conspiracy beliefs at the second wave. Likewise, a higher level of conspiracy beliefs at the first wave predicted a within‐person increase in the sense of precarity at the second wave. However, neither of these longitudinal effects was found between the second and the third wave of the study. The only two within‐person effects we found between the second and the third wave of the data collection, besides autoregressive effects, were that within‐person deviations in participants' trait‐level mean of the sense of precarity positively predicted subsequent within‐person deviations in conspiracy beliefs, and that a within‐person increase in conspiracy beliefs was predictive of subsequent within‐person decrease in trust.

Lastly, we also used this model to estimate the three indirect paths tested in the AR‐CLPM models above. First, we tested the presumed ordered longitudinal mediation effect with precarity as the predictor (X), institutional trust as the mediator (M) and conspiracy beliefs (Y) as the outcome; however, it was not statistically significant, *b* = 0.001, 95% CI [−0.004, 0.005], *p* = .73. Likewise, neither the opposite mediation path (X: conspiracy beliefs, M: institutional trust, Y: precarity), *b* = 0.001, 95% CI [−0.015, 0.017], *p* = .88, nor the third alternative model (X: institutional trust, M: conspiracy beliefs, Y: precarity), *b* = −0.009, 95% CI [−0.030, 0.012], *p* = .42, was statistically significant. Based on this, the aforementioned preregistered AR‐CLPM analyses supporting our final alternative mediation model seem to be driven primarily by stable between‐person differences, rather than within‐person longitudinal changes in the variables of interest.

## DISCUSSION

The current study aimed to longitudinally extend findings about the mediating role of institutional trust in the relationship between precarity and CBs (Adam‐Troian et al., 2023; Adamus et al., 2024). This previous correlational research suggested that the higher individuals' levels of experienced precarity are, the more likely they would adhere to CBs, and that this relationship would be mediated by institutional distrust. The current findings compellingly show that all three phenomena are tightly linked, but the relationships are not as straightforward as cross‐sectional studies had previously reported. The picture revealed here is different and perhaps even more troubling than the one provided by the original studies. Specifically, the results point to an intricate nature of the relationship between CBs, institutional trust and the sense of precarity. They demonstrate that the variables are intrinsically intertwined and, with time, may reinforce their aversive effects on one another at either the individual or the population level. A more fine‐grained analysis (RI‐CLPM) shows that the evidence for the mediation model may be driven by the stable between‐person differences rather than within‐person temporal changes. Similar difficulties with replicating the original Adam‐Troian et al. (2023) model were recently reported by Marques et al. (2025). Our exploratory analyses that did not focus on specific directional hypotheses reveal two separate within‐person patterns that firstly link conspiracy beliefs with the sense of precarity and, secondly, institutional trust with conspiracy beliefs. These additional patterns of potential temporal loops offer a new perspective on the previous findings and suggest an interesting future research avenue: it is possible that apart from the between‐person differences, the original results as well as our AR‐CLPM findings may be driven by distinct but coinciding mechanisms masked at the population level as a single effect or that there exists a yet unrecognized driver behind all the effects.

The paper's contribution could thus be seen as three‐pronged. First, it provides compelling evidence about the temporal direction of the relationships between the phenomena in question that contradicts correlational results and challenges the associated intuitive interpretations. Second, based on the findings that show how tightly institutional trust and CBs are linked in time, it warrants a closer scrutiny of the nexus between the two variables that could advance our understanding of the multiple consequences CBs may have in the social‐psychological and political realm. Finally, the results persuasively point to a more general need for conceptual longitudinal replications of well‐established findings to test whether they hold when approached with a more rigorous methodology. Changing the temporal perspective from cross‐sectional to longitudinal could considerably augment our understanding of social‐psychological realities in which both conspiracy beliefs and suspicions of institutions could flourish.

Despite failing to fully replicate the original findings (Adam‐Troian et al., 2023; Adamus et al., 2024), the current paper sends a clear methodological message to the scholarly community: in research on CBs, it is not sufficient to capture snapshots using cross‐sectional studies. Instead, to obtain a comprehensive understanding of the processes behind CBs and the full spectrum of their real‐life ramifications, it seems inevitable to turn towards longitudinal analyses. This is so because, unlike cross‐sectional studies, the current AR‐CLPM results corroborate the view that the sense of precarity *follows* adherence to CBs rather than *precedes* it. Nevertheless, our RI‐CLPM analysis suggests that at the within‐person level and over a longer time frame, this temporal pattern could actually turn out to be bidirectional. As troubling as the revealed directional patterns may seem, they are convergent with the most recent research, which increasingly employs a longitudinal and dynamic scholarly perspective on the study of CBs. For instance, our present findings are in line with results about the associations between economic anxiety – a concept broader than precarity – and CBs. The study by Adamus et al. (2025) revealed that, in temporal terms, the subjective appraisal of individual and country‐level economic situations can be seen as a consequence of CBs rather than their cause. Similarly to the current study, Adamus et al. (2025) indicate that the more people adhere to CBs, the more pessimistic their views of the economic situation may become. Concurrently, instead of being a cause, CBs could be seen as a consequence of diverse anti‐democratic sentiments (Thomas et al., 2025). This is particularly interesting in the current geopolitical context, as economic anxiety or a general sense of disillusionment with the economy and democracy are known to be relevant drivers of political preferences and voting support for populist politicians and parties (Krouwel et al., 2017; Papaioannou et al., 2024).

Recent results could also shed light on our findings about the bidirectional pattern of relationships between CBs and institutional (dis)trust legible in both AR‐CLPM and RI‐CLPM analyses. The lack of trust in democratic institutions could be seen as a reflection of discontent with political elites and the liberal‐democratic political system. The general sense of disappointment with politics and politicians may thus pave the way for increased adherence to CBs, which, in turn, may lead to even more skepticism about the functioning of democracy and its institutions as expressed by low levels of institutional trust. It is therefore plausible that people who become suspicious of institutions up to the point of being conspiracist may fall into a self‐enhancing loop of distrust and assigning nefarious intentions to economic and political elites, or – more generally – members of the out‐groups they consider to be powerful and threatening. This could contribute to the formation of the *conspicuous* (conspiracist+suspicious) mindset – a situation in which distrust and CBs become so tightly intertwined, it may be challenging to disentangle them. Therefore, we should perhaps pursue a closer examination of the nexus between CBs and distrust, and investigate the possible existence of the mindset as a reflection of complex anti‐democratic tendencies that win people's support by feeding on and further fuelling their disappointment. If it exists, the mindset could be seen as an expression of deeper underlying social‐psychological and political attitudes that in their essence are anti‐democratic, such as autocracy or anarchy (Papaioannou et al., 2024), or serve as a justification of these anti‐democratic sentiments (van Prooijen, 2018). The true roots of those sentiments, however, may need to be sought elsewhere.

From a broader perspective, our study may provide insights into the extent to which modern societies are vulnerable to the spread of both CBs and institutional distrust and their potential adverse consequences. The spread or ignition of the conspicuous mindset could contribute to more pessimistic appraisals of economic circumstances, and economic disappointment is among the most relevant drivers of popular votes (Krouwel et al., 2017). As a consequence, conspiracy theorists may also fall into a trap of CBs, suspicions, and – as the current results show – pessimistic appraisal of their living conditions. When people become more and more skeptical about their social, political and economic circumstances, they may more easily fall prey to interference by populists or foreign agents, who may thereby manipulate the polls. By using the CBs and sowing institutional distrust as a form of information warfare – and the sense of economic insecurity as its corollary – opportunistic agents could hope to muster greater political support and expect that people's insecurity, frustration and disappointment would translate into voting preferences (Petersen et al., 2021).

Finally, this study is not free from limitations. Although our data provide valuable information about the temporal relationships in question and come from an understudied country in Central Europe, the study was conducted in a single cultural context. It is possible that in other cultural contexts, the relationships may not hold or the directional patterns of the relationships could be different. Fortunately, a recent longitudinal study on the link between trust and CBs (van Prooijen et al., 2022) corroborates our results and lends additional credence to our findings. Nevertheless, it would be beneficial if future research could examine these associations with longitudinal data, preferably stemming from both WEIRD and non‐WEIRD contexts globally. Additionally, we acknowledge that our operationalizations of variables relevant to the model tested in this paper might be limited. Compared to the original Adam‐Troian et al. (2023) paper, we employed a different set of CBs, and our operationalization of precarity is also distinct. Precarity is a multidimensional phenomenon that captures various aspects of existential insecurity, such as relative deprivation, marginalization, exclusion, low prestige and limited possibilities of upward social mobility (Standing, 2011). In this study, we focus on only one of the many facets of precarity–financial insecurity–but we need to remember that precarity also encompasses low job security as well as the sense of physical threats to one's safety. Moreover, CBs analysed in the present study were exclusively associated with people's views about the COVID‐19 pandemic. The Adam‐Troian et al. (2023) paper, whose results we attempted to replicate, focused on more context‐independent CBs about electoral fraud that could have even stronger relationships with institutional trust. While these differences in operationalizations may be responsible for the strengths or even directions of relationships, our longitudinal results, in general, corroborate and accentuate the original correlational ones by providing persuasive evidence that all three variables are associated regardless of the specific operationalizations. This, in turn, shows that the original Adam‐Troian et al. (2023) paper taps into a more universal pattern that goes beyond specific CBs or approximations of precarity. What the current paper adds is another layer of interpretation that transcends correlational observations and allows us to notice the temporal dimension of the findings. Specifically, it allows us to see that while institutional distrust, CBs and precarity are all associated, in temporal terms, distrust and CBs may precede the sense of precarity. Finally, despite our efforts, we were unable to access any longitudinal dataset that would include repeated measures of both specific conspiracy beliefs and conspiracy mentality (Imhoff et al., 2022). Future research should thus endeavour to carefully disentangle the two concepts by consistently capturing them at different time points. This could allow scholars to gain deeper insights into the nature of the relationships and potentially diverse ramifications that CBs and conspiracy mentality may have in the social‐psychological, economic and political domains.

## CONCLUSIONS

Despite the limitations, our study provides important insights into the consequences CBs and institutional distrust may have for modern societies. The study of the nexus between the two is far from being a sophisticated field of abstract considerations and should no longer be treated as a fringe cognitive phenomenon. We now clearly see that they pose a challenge to the resilience, cohesion and health of societies – and a very real one, with activists, scientists, medics and even celebrities facing vicious attacks all over the world because they have once made some unpopular claim. The mindset thus has real and gloomy consequences for entire societies and for the individuals who endorse it as well as those who become its targets. In times when CBs are being used to manipulate popular votes and being weaponized in so‐called hybrid or hot wars, the threat they pose should not be underestimated. We need to be prepared to combat CBs not only as a cognitive bias but as a real threat to the stability of societies. Recognizing this is not just a scientific imperative, but a societal necessity, for in their spread lies a quiet erosion of trust, reason and democratic resilience.

## AUTHOR CONTRIBUTIONS

**Magdalena Adamus:** Conceptualization; supervision; writing – original draft. **Jakub Šrol:** Conceptualization; methodology; formal analysis; writing – review and editing. **Eva Ballová Mikušková:** Conceptualization; writing – review and editing. **Jais Adam‐Troian:** Conceptualization; writing – review and editing. **Maria Chayinska:** Writing – original draft; supervision; conceptualization.

## Supporting information


Data S1.

## Data Availability

All materials, raw data, and analysis scripts used in this study are available at: https://osf.io/pqk9z/overview.
