# Supplementary material for: Longitudinal analysis shows possible distinct patterns of associations between conspiracy beliefs and either institutional distrust or the sense of precarity
Source: Br J Soc Psychol. 2025 Dec 15;65(1):e70036. doi: 10.1111/bjso.70036 (PMC12704043; doi:10.1111/bjso.70036)
Supplement: Supplementary file 1 — Data S1. [file BJSO-65-0-s001.docx]

**Supplementary material**

**SECTION A**

**Attrition check**

Following the procedure from Coelho et al. (2022), in order to estimate whether attrition systematically influenced the variables included in our models, we conducted a Poisson regression in which we used following variables from the first wave of the study as predictors of the number of subsequent waves completed by our participants: institutional trust, precarity, conspiracy beliefs, age, gender (2 categories: man/woman), education (2 categories: elementary education or high school education without a diploma / high school with a diploma or any tertiary education), and subjective socio-economic status.

The results showed that neither of our three key variables of interest (i.e., institutional trust, the sense of precarity, and conspiracy beliefs) were not significantly associated with the number of subsequent waves finished: institutional trust (*B* = –0.010, *p* = .40, 95%CI [–0.034, 0.014]), precarity (*B* = –0.007, *p* = .77, 95%CI [–0.053, 0.039]), conspiracy beliefs (*B* = –0.038, *p* = .20, 95%CI [–0.095, 0.020]). However, while gender (*B* = 0.017, *p* = .70, 95%CI [–0.066, 0.100]) and subjective socio-economic status (*B* = –0.016, *p* = .27, 95%CI [–0.045, 0.013]) were likewise not significant predictors, both age (*B* = 0.009, *p* < .001, 95%CI [0.006, 0.011]) and education level (*B* = 0.223, *p* < .001, 95%CI [0.128, 0.319]) were positively associated with the number of subsequent waves completed. These results suggest that while we have collected a quota sample of Slovak population in the first wave of the study, our final sample underrepresents people of younger age and lower education. While this poses limits to the generalizability of our findings, we believe it does not affect our main conclusions as neither of the three key variables (institutional trust, precarity or conspiracy beliefs) was significantly associated with the number of completed subsequent waves of the study.

**SECTION B**

**Robustness check with the consistent measure of institutional trust across waves**

As a robustness check, we replicated our two main mediation models (both the retained autoregressive cross-lagged panel model and the random-intercept cross-lagged panel model from the main manuscript) while using the consistent set of eight items to reflect trust in institutions across the three waves. The data, codebook and script for these analyses are available at: https://osf.io/pqk9z/. First, we tested the retained alternative autoregressive cross-lagged panel model (X: institutional trust, M: conspiracy beliefs, Y: precarity, see Fig. S1). The model showed a very good fit to the data: χ^2^(13) = 43.17, *p* < .001, CFI = .99, TLI = .99, RMSEA = .050, SRMR = .022. As in the main manuscript, the alternative indirect longitudinal path estimated in this model (b1*c4) was significant, estimate = –0.006, 95% CI [–0.011, –0.001], standardized = –.015, *p* = .013. As can be seen from the figure, all of the paths estimated in the model are consistent with the model presented in our manuscript, providing evidence for the robustness of our findings.


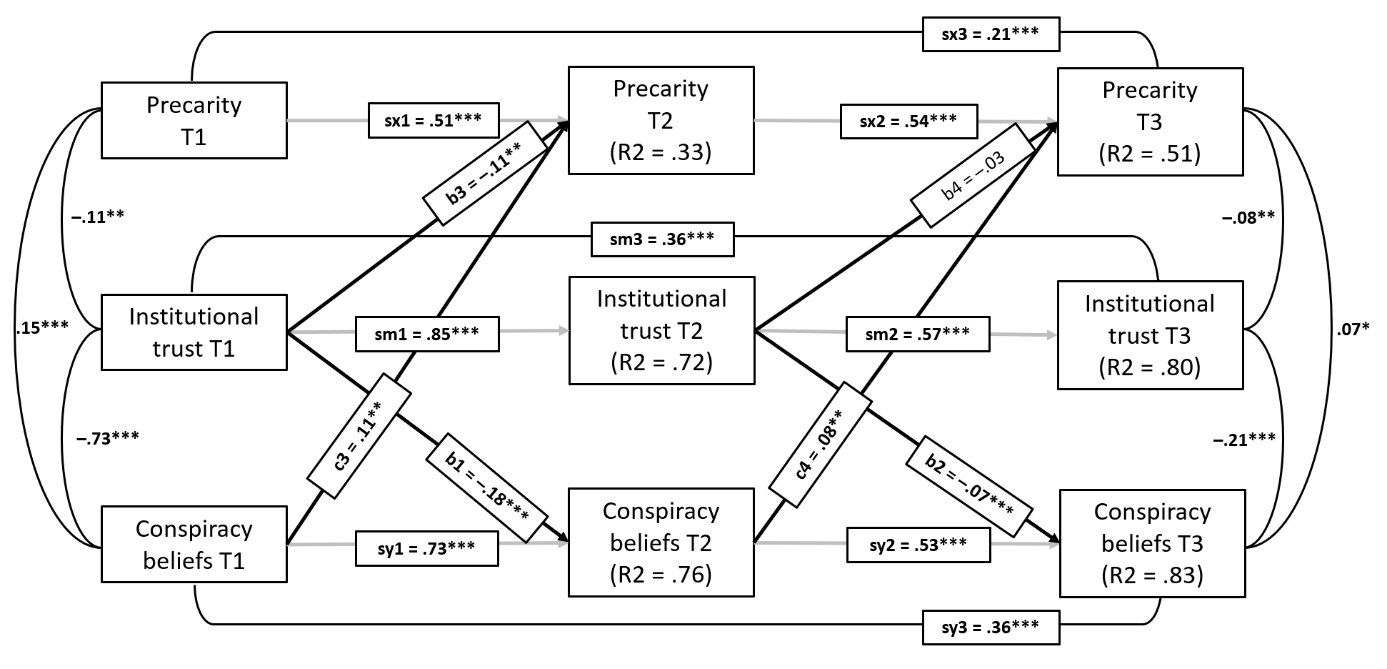


**Fig. S1. Autoregressive mediation model with only alternative paths between precarity, institutional trust. and conspiracy beliefs estimated.** The figure presents standardised estimates and their statistical significance. * *p* < .05, ** *p* < .01, *** *p* < .001

**Random intercept cross-lagged panel model**

Next, we also recalculated the random intercept cross-lagged panel model using only the eight items to consistently measure trust in institutions across the three study waves. The model, estimated using R package *lavaan* (Rosseel, 2012) with standard errors based on 2,000 bootstrap samples, showed a good fit to the data according to most of the indicators, χ2(3) = 28.85, *p* < .001, CFI = .99, TLI = .96, RMSEA = .097, SRMR = .027. As can be seen from Fig. S2, at the between-person level, the random intercepts for our three variables were significantly correlated. Specifically, completely standardized coefficients for the covariances showed that precarity was associated with lower trust (*r* = –.22) and higher belief in conspiracy theories (*r* = .25), and that the latter two variables were very strongly negatively correlated (*r* = –.79). On the level of within-person associations, we observe the same paths as in the model presented in the main manuscript, with the one exception. While within-person deviation from the trait-level mean of institutional trust at the second wave of the study did not predict subsequent within-person deviation in trust at the third wave in the model presented in the main manuscript, this autoregressive path was significant here.

More importantly, however, all of the indirect paths tested in the model were insignificant, consistently with the main manuscript. Specifically, the presumed ordered longitudinal mediation effect with precarity as the predictor (X), institutional trust as the mediator (M), and conspiracy beliefs (Y) as the outcome was not statistically significant, *b* = –0.001, 95% CI [–0.025, 0.009], *p* = .97. Likewise, neither the opposite mediation path (X: conspiracy beliefs, M: institutional trust, Y: precarity), *b* = 0.002, 95% CI [–0.015, 0.046], *p* = .91, nor the third alternative path (X: institutional trust, M: conspiracy beliefs, Y: precarity), *b* = –0.008, 95% CI [–0.035, 0.012], *p* = .53, were statistically significant. Thus, all of the results are substantially consistent with the conclusions presented in the main manuscript.


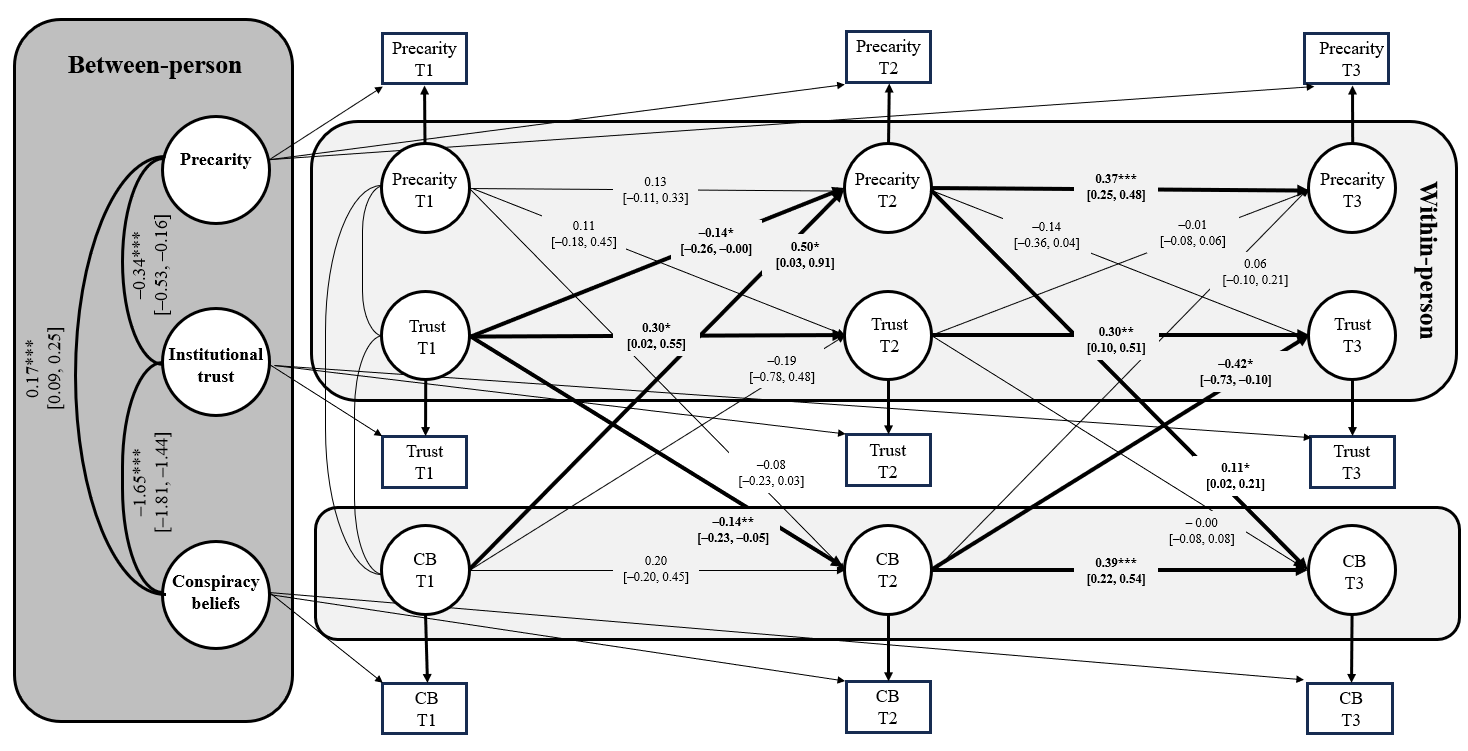


**Fig. S2. Random intercept cross-lagged panel model of the relationships between precarity, institutional trust, and conspiracy beliefs.** Values for paths represent unstandardized coefficients along with their 95% confidence intervals. CB = conspiracy beliefs. Significant paths (*p* < .05) are presented in bold. * *p* < .05, ** *p* < .01, *** *p* < .001

**REFERENCES**

Coelho, P., Foster, K., Nedri, M., & Marques, M. D. (2022). Increased belief in vaccination conspiracy theories predicts increases in vaccination hesitancy and powerlessness: Results from a longitudinal study. *Social Science & Medicine, 315*, 115522. https://doi.org/10.1016/j.socscimed.2022.115522
